# Manufacturing of breathable, washable, and fabric-integrated squid skin-inspired thermoregulatory materials

**DOI:** 10.1063/5.0169558

**Published:** 2024-10-01

**Authors:** Sanghoon Lee, Erica M. Leung, Mohsin Ali Badshah, Aleksandra Anna Strzelecka, Alon A. Gorodetsky

**Affiliations:** 1 Department of Chemical and Biomolecular Engineering, University of California, Irvine, California 92697, USA; 2 Department of Materials Science and Engineering, University of California, Irvine, California 92697, USA

## Abstract

Advanced thermal management technologies represent an important research frontier because such materials and systems show promise for enhancing personal physiological comfort and reducing building energy consumption. These technologies typically offer the advantages of excellent portability, user-friendly tunability, energy efficiency, and straightforward manufacturability, but they frequently suffer from critical challenges associated with poor breathability, inadequate wash stability, and difficult fabric integration. Within this broader context, our laboratory has previously developed heat-managing composite materials by drawing inspiration from the color-changing skin of the common squid. Herein, we describe the design, fabrication, and testing of breathable, washable, and fabric-integrated variants of our composite materials, which demonstrate state-of-the-art adaptive infrared properties and dynamic thermoregulatory functionalities. The combined findings directly advance the performance and applications scope of our bioinspired thermoregulatory composites and ultimately may guide the incorporation of desirable multifunctionality into other wearable technologies.

## INTRODUCTION

Advanced thermal management technologies, such as wearable materials, personal cooling/heating devices, and portable ventilation systems, represent an important research frontier because of their potential for enhancing personal physiological comfort and reducing building energy consumption.^1–19^ In particular, wearable materials and systems, i.e., engineered textiles or membranes, have proven adept at locally regulating radiative, conductive, and/or convective heat exchange between the human body and its surrounding environment.^7–19^ Generally, such materials and systems can offer the advantages of excellent portability, user-friendly tunability, energy efficiency, and straightforward manufacturability.^7–19^ However, despite much recent progress, wearable materials and systems still frequently suffer from critical challenges associated with poor breathability, inadequate wash stability, and difficult fabric integration.^7–17^ Therefore, there still exists a need for the development and validation of robust general methodologies that can surmount the aforementioned challenges, which often preclude practical applications for many wearable technologies.

**FIG. 1.**
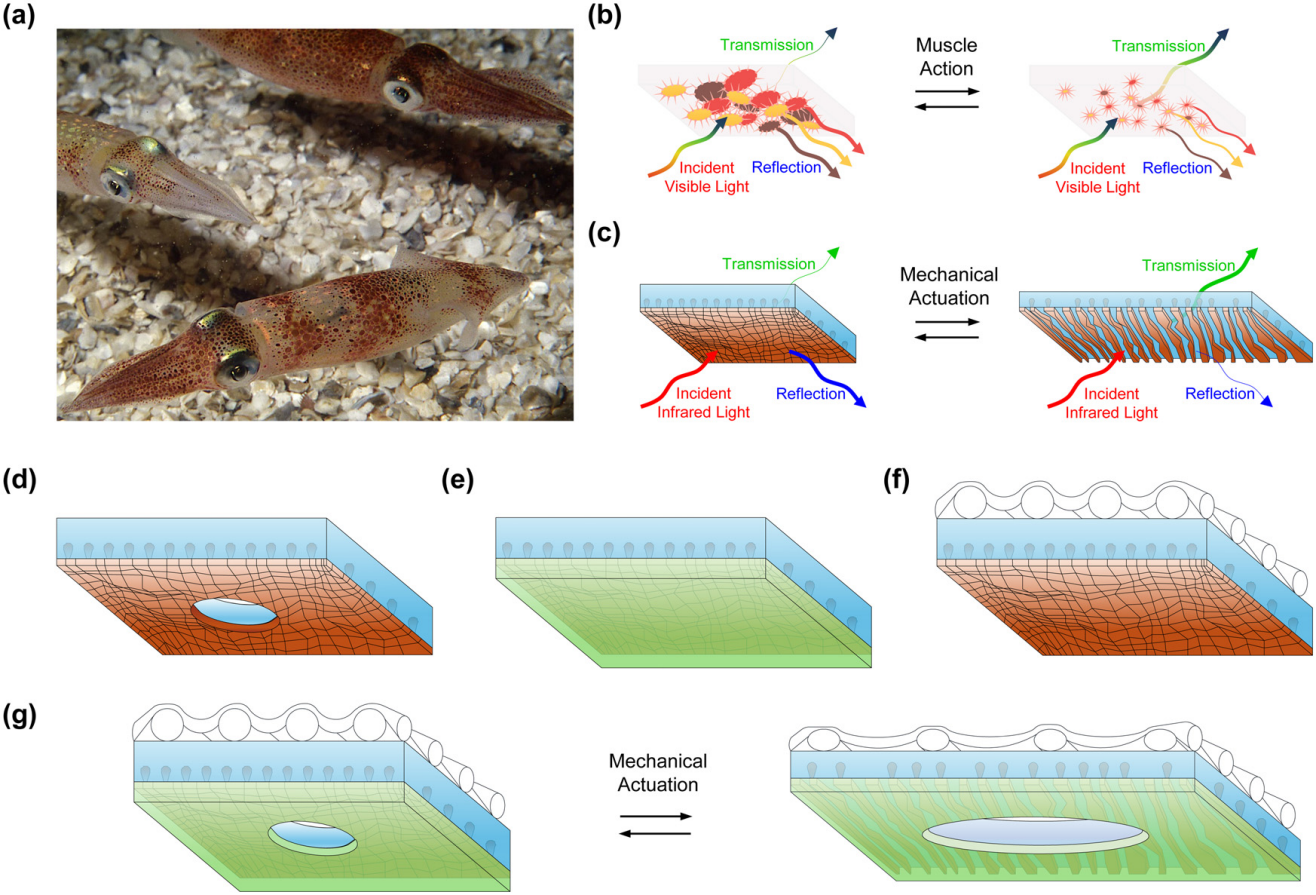
Squid skin-inspired design of breathable, washable, and fabric-integrated adaptive infrared (and dynamic thermoregulatory) composite materials. (a) A digital camera image of multiple live longfin inshore squids and their color-changing skin. (b) A schematic of a squid skin layer containing embedded chromatophore organs, which transition between expanded (left) and contracted (right) states as a result of muscle action. The layer adaptively modulates the transmission and reflection of visible light. (c) A schematic of a standard thermoregulatory composite material consisting of polymer matrix containing embedded metal islands, which transition between abutted (left) and separated (right) states upon the application of strain. The composite material adaptively modulates the transmission and reflection of infrared light (and the flow of heat). (d) A schematic of a breathable perforated composite material. (e) A schematic of a washable encapsulated composite material. (f) A schematic of a wearable fabric-integrated composite material. (g) A schematic of a breathable perforated, washable encapsulated, and wearable fabric-integrated composite material before (left) and after (right) the application of strain. The multifunctional composite material's polymer matrix contains embedded metal islands, which transition between abutted (left) and separated (right) states upon the application of strain. Note that the picture in (a) is reproduced with permission from D. Kenney, *New Genetic Editing Powers Discovered in Squid* (Marine Biological Laboratory, 2020). Copyright 2020 Roger Hanlon.^23^

Within the context of thermal management technologies, our laboratory has developed heat-managing composite materials by drawing inspiration from the color-changing skin of the longfin inshore (and related) squid, as illustrated in Fig. 1(a).^20–26^ Specifically, we have considered squid skin layers containing embedded organs called chromatophores, which transition between expanded and contracted states (upon muscle action) and, thus, modulate the transmission and reflection of visible light, as illustrated in Fig. 1(b).^20–22^ Accordingly, we have engineered composite materials consisting of styrene–ethylene–butylene–styrene (SEBS) polymer matrices containing embedded copper (Cu) metal domains, which transition between abutted and separated states (upon the application of strain) and, thus, modulate the transmission and reflection of infrared light, as illustrated in Fig. 1(c).^24–26^ We have shown that such materials feature advantageous mechanical properties, as exemplified by their Young's moduli of ∼1 to ∼2 MPa and breaking strains of ∼700% to ∼1300% (supplementary material Table 1).^24–26^ We have also demonstrated that these materials not only modulate their computationally predictable reflectances and transmittances by > 30% but also regulate heat fluxes of > 30 W/m^2^ upon mechanical actuation (supplementary material Tables 2 and 3).^24–26^ We have, moreover, proven that the materials can be scalably manufactured in different form factors via modular procedures and are, thus, readily integrated into proof-of-principle sleeve-type wearable systems.^24–26^ However, in the previous efforts, we have not explored improving our composite materials' breathabilities, wash stabilities, and fabric compatibilities, which are essential considerations for all wearable applications.^24–26^

Herein, we substantially advance our previous efforts by developing breathable, washable, and fabric-integrated variants of our wearable squid skin-inspired thermoregulatory materials. First, we prepare and evaluate perforated composites [Fig. 1(d)] that feature air and water vapor permeabilities rivaling those of common cotton. Second, we prepare and evaluate encapsulated composites [Fig. 1(e)] that exhibit wash cycle stabilities comparable to those of commercial fabrics. Third, we prepare and evaluate mesh-integrated composites [Fig. 1(f)] that possess fabric compatibilities analogous to those of typical laminates. Finally, we prepare and evaluate perforated, encapsulated, and mesh-integrated composites [Fig. 1(g)], which merge all of our other modified composites' advantageous attributes. Notably, the different described composites generally maintain their mechanical characteristics, fundamental operating modes, adaptive infrared properties, cycling stabilities, and dynamic thermoregulatory functionalities. Therefore, the reported methodology not only directly advances the functionalities and application scopes of our wearable thermoregulatory materials but may also help guide analogous improvements for other wearable technologies.

## RESULTS

### Preparation and evaluation of breathable composite materials

**FIG. 2.**
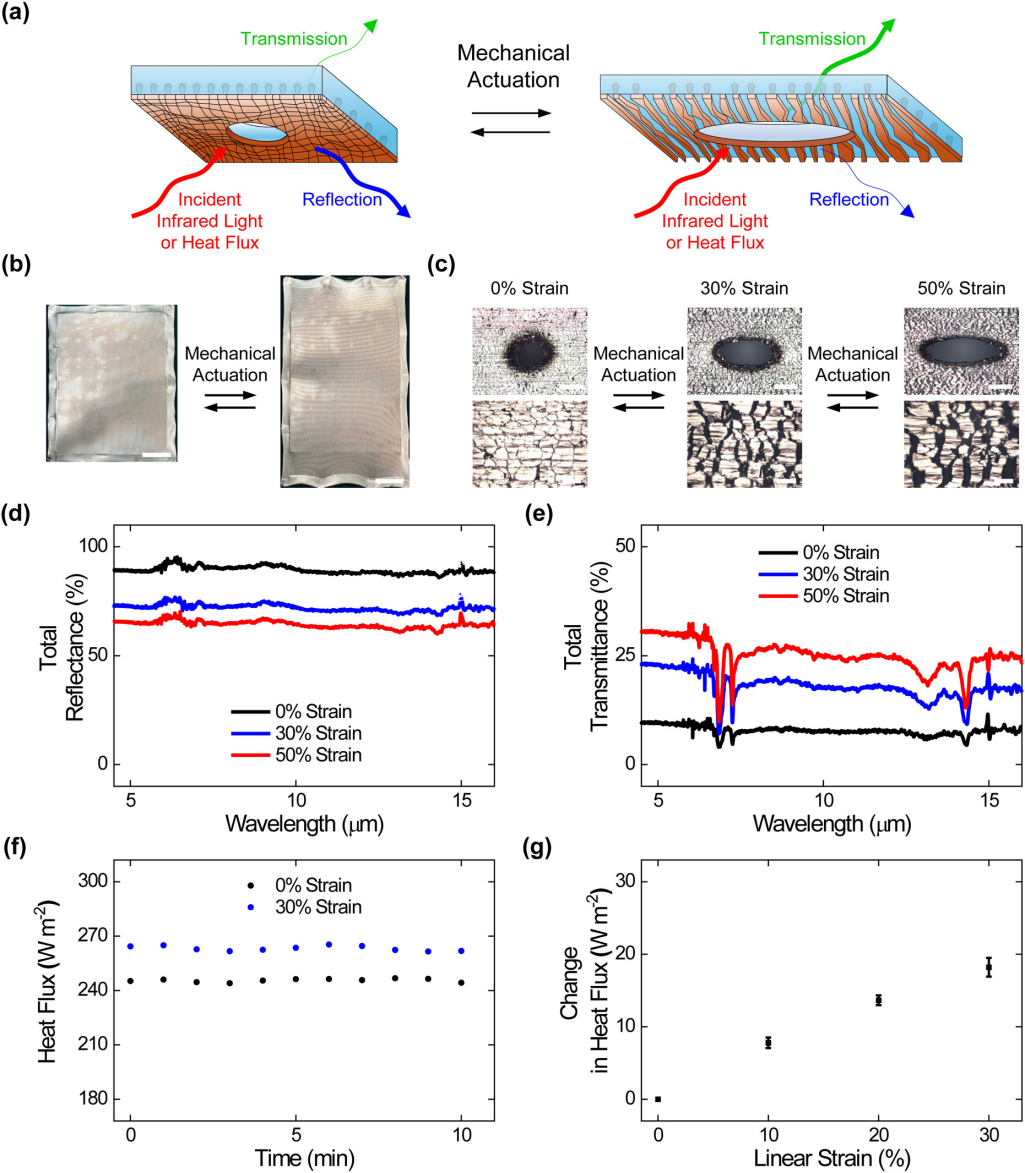
Surface morphologies, adaptive infrared properties, and dynamic thermoregulatory functionalities of the perforated composite materials. (a) A schematic of a perforated composite material that undergoes changes in surface microstructure and, thus, adaptively modulates infrared light and heat upon mechanical actuation. (b) Digital camera images of perforated composites under applied strains of 0% (left) and 50% (right). The composites feature areas of ≥560 cm^2^. The scale bars are 4 cm. (c) High (top) and low (bottom) magnification optical microscopy images of the perforated composites under applied strains of 0% (left), 30% (middle), and 50% (right). The images show that embedded metal islands transition between abutted and separated states upon the application of strain. The scale bars are 100 *μ*m for the top images and 20 *μ*m for the bottom images. (d) The total infrared reflectance spectra obtained for the perforated composites under applied strains of 0% (black), 30% (blue), and 50% (red). (e) The total infrared transmittance spectra obtained for the perforated composites under applied strains of 0% (black), 30% (blue), and 50% (red). (f) The plot of the time-dependent heat fluxes obtained for the perforated composites under applied strains of 0% (black) and 30% (blue). (g) The average steady-state heat flux changes measured for the perforated composites under applied strains of 0%, 10%, 20%, and 30%. The error bars in (g) represent standard deviation of the mean.

We began our efforts by preparing perforated composite materials and characterizing them without and with mechanical strain, as illustrated in Figs. 1(d) and 2(a). To this end, we fabricated large-area composites featuring arrayed holes in the SEBS matrix/overlaid Cu layer by modifying reported protocols, as illustrated in supplementary material Fig. 1(a), and we interrogated the perforated composites with digital camera imaging, optical microscopy, and tensile testing (see Methods for additional information). The digital camera images revealed that the obtained perforated composites featured areas of ≥560 cm^2^, were covered by ∼200 *μ*m holes with edge-to-edge separations of ∼1 mm, and were readily deformed via applied strain [Fig. 2(b) and supplementary material Fig. 1(b)]. The engineering stress vs strain curves and corresponding camera images indicated that the perforated composites featured elastomeric behavior, with Young's moduli of ∼0.6 MPa and breaking strains of ∼700% comparable to those of analogous composites without perforation (supplementary material Fig. 2 and supplementary material Table I).^24–28^ The local optical microscopy images showed that the perforated composites' metal layers contained round holes surrounded by abutting Cu domains without any applied strain but contained oval holes surrounded by separated Cu domains upon the application of strain, indicating that the previously reported operating mechanism was maintained [Fig. 2(c)].^24–26^ These experiments demonstrated the straightforward fabrication of perforated composite materials with large areas, robust mechanical properties, and reconfigurable surface microstructures.

We next assessed the adaptive infrared properties of the perforated composite materials, as shown in Fig. 2(a). For this purpose, we characterized the composite materials with Fourier transform infrared (FTIR) transmittance and reflectance spectroscopy according to established protocols (see Methods for additional information). The total infrared reflectance spectra obtained for the composites indicated average values that progressively decreased from ∼88% ± 1% to ∼72% ± 1% to ∼64% ± 1% under applied strains of 0%, 30%, and 50%, respectively [Fig. 2(d)]. The total infrared transmittance spectra obtained for the composites indicated average values that progressively increased from ∼8% ± 1% to ∼17% ± 1% to ∼25% ± 1% under applied strains of 0%, 30%, and 50%, respectively [Fig. 2(e)]. Here, the average reflectance and transmittance changes observed for the perforated composite materials were slightly smaller than those reported for analogous composite materials without any perforation (see the direct comparison in supplementary material Table 2), presumably because of additional transmission of infrared radiation through the arrayed holes.^25^ Notably, the perforated composites' average reflectance and transmittance modulation remained relatively consistent even after 1000, 5000, and 10 000 mechanical actuation cycles (supplementary material Fig. 3). These experiments showed that our composite materials' user-tunable infrared-reflecting and infrared-transmitting functionalities were generally maintained even after perforation.

We, in turn, assessed the dynamic thermoregulatory functionalities of the perforated composite materials, as shown in Fig. 2(a). For this purpose, we characterized the composite materials with calibrated heat flux measurements on a sweating guarded hot plate (SGHP) according to established protocols (see Methods for additional information). The plot of the time-dependent heat fluxes obtained for a representative perforated composite indicated a value of ∼245 W/m^2^ under an applied strain of 0% and a value of ∼263 W/m^2^ under an applied strain of 30% [Fig. 2(f)]. The average steady-state heat flux changes measured for such composites could be readily adjusted between ∼8 ± 1 and ∼18 ± 2 W/m^2^ by the applied strain [Fig. 2(g)]. Here, the heat flux changes observed for the perforated composite materials were again slightly smaller than those reported for analogous composites without any perforation (see the direct comparison in supplementary material Table 3), presumably because of the flow of heat through the arrayed holes.^25^ These experiments showed that our composite materials' user-controllable heat-managing functionalities were generally maintained even after perforation.

We last comparatively benchmarked the air and water vapor permeabilities of the perforated composite materials. To achieve this goal, we characterized the composite materials with air and water vapor permeability measurements according to standard commercial protocols (see Methods for additional information). The air and water vapor permeabilities obtained for the perforated composites were ∼81 ± 6 ft^3^/ft^2^/min and ∼840 ± 30 g/m^2^/day, respectively (supplementary material Table 4). In contrast, the air and water vapor permeabilities obtained for analogous composites without perforation were 0 ft^3^/ft^2^/min and 0 g/m^2^/day, respectively (supplementary material Table 4). Comparatively, the air and water vapor permeabilities previously reported for typical cotton fabrics were ∼66 ft^3^/ft^2^/min and ∼367 g/m^2^/day, respectively (supplementary material Table 4).^29,30^ These experiments demonstrated that our perforated composite materials featured fabric-like breathabilities, which is a critical consideration for wearable applications.

### Preparation and evaluation of washable composite materials

**FIG. 3.**
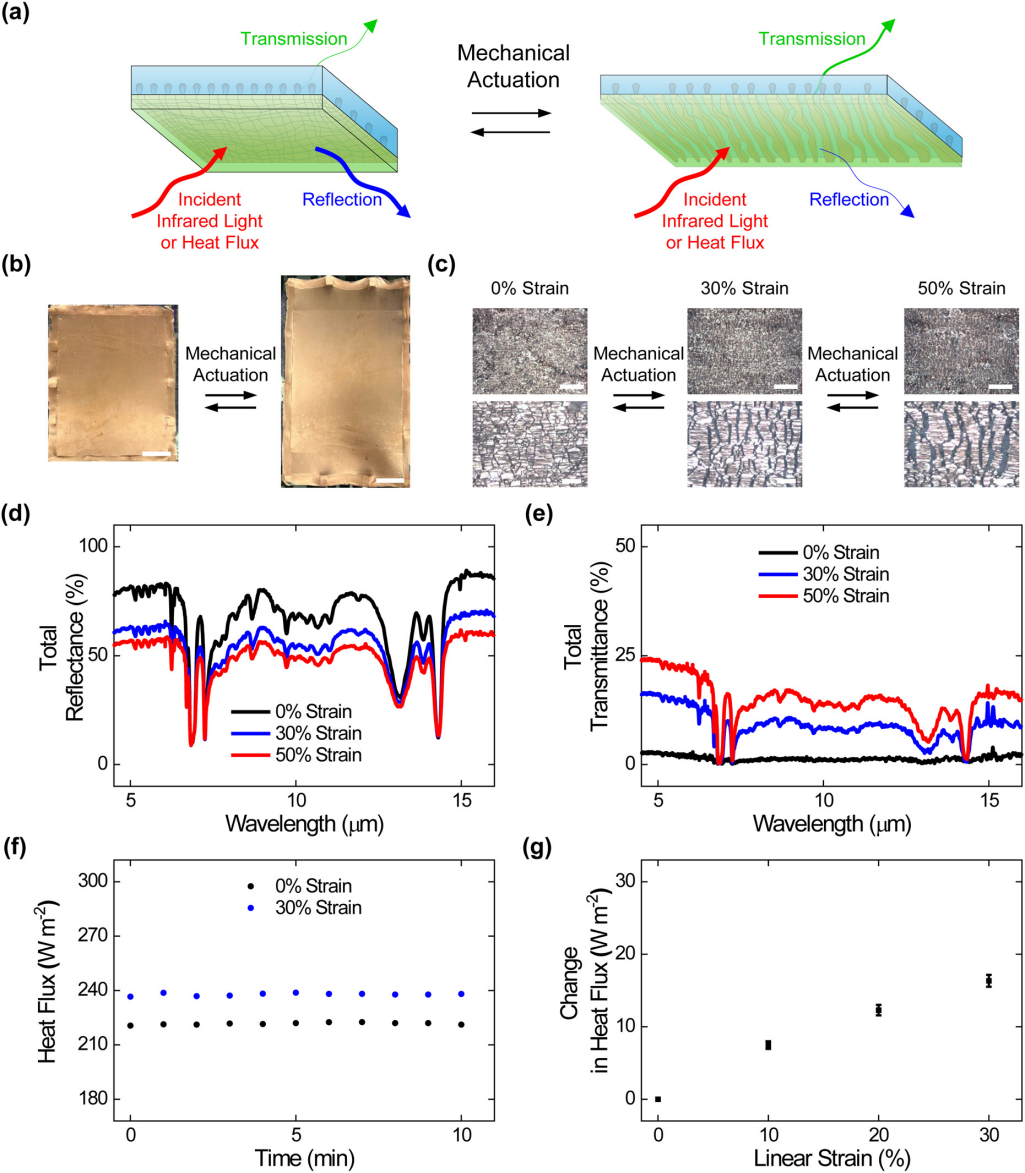
Surface morphologies, adaptive infrared properties, and dynamic thermoregulatory functionalities of the encapsulated composite materials. (a) A schematic of an encapsulated composite material that undergoes changes in surface microstructure and, thus, adaptively modulates infrared light and heat upon mechanical actuation. (b) Digital camera images of encapsulated composites under applied strains of 0% (left) and 50% (right). The composites feature areas of ≥560 cm^2^. The scale bars are 4 cm. (c) High (top) and low (bottom) magnification optical microscopy images of the encapsulated composites under applied strains of 0% (left), 30% (middle), and 50% (right). The images show that embedded metal islands transition between abutted and separated states upon the application of strain. The scale bars are 100 *μ*m for the top images and 20 *μ*m for the bottom images. (d) The total infrared reflectance spectra obtained for the encapsulated composites under applied strains of 0% (black), 30% (blue), and 50% (red). (e) The total infrared transmittance spectra obtained for the encapsulated composites under applied strains of 0% (black), 30% (blue), and 50% (red). (f) The plot of the time-dependent heat fluxes obtained for the encapsulated composites under applied strains of 0% (black) and 30% (blue). (g) The average steady-state heat flux changes measured for the encapsulated composites under applied strains of 0%, 10%, 20%, and 30%. The error bars in (g) represent standard deviation of the mean.

We continued our efforts by preparing encapsulated composite materials and characterizing them without and with mechanical strain, as illustrated in Figs. 1(e) and 3(a). To this end, we fabricated large-area composites featuring an additional SEBS encapsulation layer that covered the abutting Cu domains, as illustrated in supplementary material Fig. 4(a), and we interrogated the encapsulated composites with digital camera imaging, optical microscopy, and tensile testing (see Methods for additional information). The digital camera images revealed that the obtained encapsulated composites featured areas of ≥ 560 cm^2^, were completely enclosed by the encapsulation layer, and were readily deformed via applied strain [Fig. 3(b) and supplementary material Fig. 4(b)]. The engineering stress vs strain curves and corresponding camera images indicated that the encapsulated composites featured elastomeric behavior, with Young's moduli of ∼1.1 MPa and breaking strains of ∼800% comparable to those of analogous composites without encapsulation (supplementary material Fig. 5 and supplementary material Table 1).^24–26^ The local optical microscopy images showed that the encapsulated composites' internal metal layers consisted of abutting Cu domains without any applied strain and consisted of separated Cu domains upon the application of strain, indicating that the previously reported operating mechanism was maintained [Fig. 3(c)].^24–26^ These experiments demonstrated the straightforward fabrication of encapsulated composite materials with large areas, robust mechanical properties, and reconfigurable surface microstructures.

We next assessed the adaptive infrared properties of the encapsulated composite materials, as shown in Fig. 3(a). For this purpose, we characterized the composite materials with FTIR transmittance and reflectance spectroscopy according to established protocols (see Methods for additional information). The total infrared reflectance spectra obtained for the composites indicated average values that progressively decreased from ∼69% ± 1% to ∼55% ± 1% to ∼48% ± 1% under applied strains of 0%, 30%, and 50%, respectively [Fig. 3(d)]. The total infrared transmittance spectra obtained for the composites indicated average values that progressively increased from ∼1% ± 1% to ∼9% ± 1% to ∼14% ± 1% under applied strains of 0%, 30%, and 50%, respectively [Fig. 3(e)]. Here, the average reflectance and transmittance changes observed for the encapsulated composite materials were smaller than those reported for analogous composites without additional encapsulation (see the direct comparison in supplementary material Table 2), presumably because of additional contributions to the strain-dependent reflectance and transmittance spectra from the overlaid SEBS-based encapsulation layer.^24–26,31,32^ Notably, the encapsulated composites' average reflectance and transmittance modulation remained relatively consistent even after 1000, 5000, and 10 000 mechanical actuation cycles (supplementary material Fig. 6). These experiments showed that our composite materials' user-tunable infrared-reflecting and infrared-transmitting functionalities were generally maintained even after encapsulation.

We, in turn, assessed the dynamic thermoregulatory functionalities of the encapsulated composite materials, as shown in Fig. 3(a). For this purpose, we characterized the composite materials with calibrated heat flux measurements on a SGHP according to established protocols (see Methods for additional information). The plot of the time-dependent heat fluxes obtained for a representative encapsulated composite indicated a value of ∼221 W/m^2^ under an applied strain of 0% and a value of ∼237 W/m^2^ under an applied strain of 30% [Fig. 3(f)]. The average steady-state heat flux changes measured for such composites could be readily adjusted between ∼7 ± 1 and ∼16 ± 2 W/m^2^ by the applied strain [Fig. 3(g)]. Here, the heat flux changes observed for the encapsulated composite materials were again smaller than those reported for analogous composites without any encapsulation (see the direct comparison in supplementary material Table 3), presumably because of added insulation by the SEBS-based encapsulation layer.^25^ These experiments showed that our composite materials' user-controllable heat-managing functionalities were generally maintained even after encapsulation.

We last comparatively benchmarked the wash stability of the encapsulated composite materials. To achieve this goal, we characterized the composite materials with digital camera imaging and FTIR spectroscopy across multiple wash/dry cycles (see Methods for additional information). Notably, the appearances of the encapsulated composites remained completely unchanged (with no loss of Cu) even after 0, 1, 10, and 20 consecutive wash/dry cycles [supplementary material Fig. 7(a)]. The average total infrared reflectance and transmittance changes obtained for the encapsulated composites remained indistinguishable from one another after 0, 1, 10, and 20 wash/dry cycles [supplementary material Figs. 7(b) and 7(c)]. In contrast, the appearances of the analogous composites without encapsulation were substantially altered after 10 wash/dry cycles and revealed nearly complete removal of the Cu layer during washing [supplementary material Fig. 8(a)]. The average total infrared reflectance and transmittance changes obtained for the analogous composites without encapsulation decreased substantially after 10 wash/dry cycles [supplementary material Figs. 8(b) and 8(c)]. These experiments demonstrated that our encapsulated composite materials could be continuously washed much like standard fabrics, which is a rare achievement for wearable dynamic thermoregulatory materials.^10–17^

### Preparation and evaluation of fabric-integrated composite materials

**FIG. 4.**
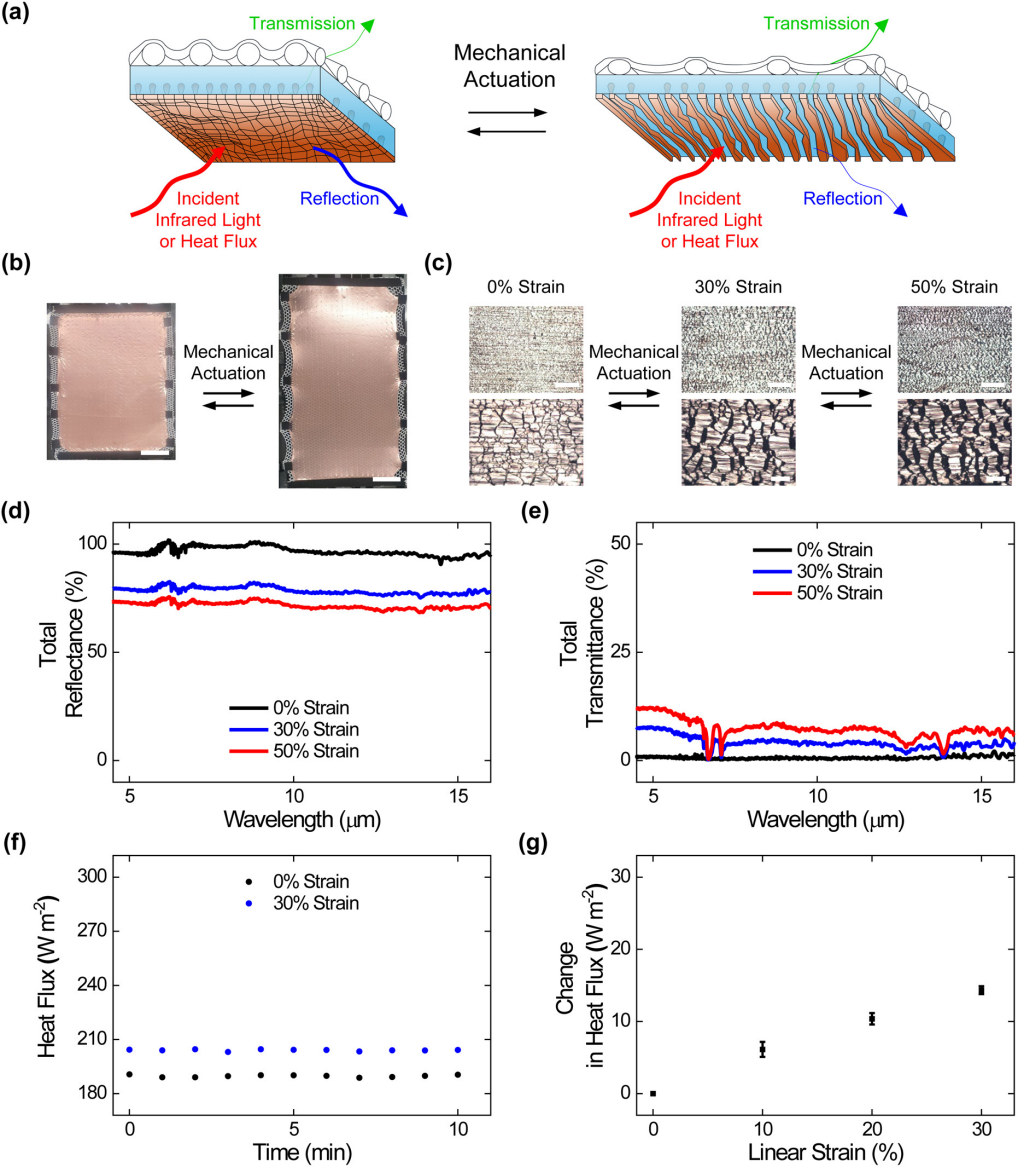
Surface morphologies, adaptive infrared properties, and dynamic thermoregulatory functionalities of the fabric-integrated composite materials. (a) A schematic of a fabric-integrated composite material that undergoes changes in surface microstructure and, thus, adaptively modulates infrared light and heat upon mechanical actuation. (b) Digital camera images of fabric-integrated composites under applied strains of 0% (left) and 50% (right). The composites feature areas of ≥560 cm^2^. The scale bars are 4 cm. (c) High (top) and low (bottom) magnification optical microscopy images of the fabric-integrated composites under applied strains of 0% (left), 30% (middle), and 50% (right). The images show that embedded metal islands transition between abutted and separated states upon the application of strain. The scale bars are 100 *μ*m for the top images and 20 *μ*m for the bottom images. (d) The total infrared reflectance spectra obtained for the fabric-integrated composites under applied strains of 0% (black), 30% (blue), and 50% (red). (e) The total infrared transmittance spectra obtained for the fabric-integrated composites under applied strains of 0% (black), 30% (blue), and 50% (red). (f) The plot of the time-dependent heat fluxes obtained for the fabric-integrated composites under applied strains of 0% (black) and 30% (blue). (g) The average steady-state heat flux changes measured for the fabric-integrated composites under applied strains of 0%, 10%, 20%, and 30%. The error bars in (g) represent standard deviation of the mean.

We continued our efforts by preparing fabric-integrated composite materials and characterizing them without and with mechanical strain, as illustrated in Figs. 1(f) and 4(a). To this end, we fabricated large-area composites adhered to a commercial mesh by modifying reported protocols, as illustrated in supplementary material Fig. 9(a), and we interrogated the mesh-integrated composites with digital camera imaging, optical microscopy, and tensile testing (see Methods for additional information). The digital camera images revealed that the obtained mesh-integrated composites featured areas of ≥560 cm^2^, were uniformly attached to the mesh, and were readily deformed via applied strain [Fig. 4(b) and supplementary material Fig. 9(b)]. The engineering stress vs strain curves and corresponding camera images indicated that the mesh-integrated composites featured elastomeric behavior with Young's moduli of ∼1.2 MPa comparable to those of the standard composites but breaking strains of ∼300% comparable to those of the standalone meshes (supplementary material Fig. 10 and supplementary material Table 1).^24–26^ The local optical microscopy images showed that the mesh-integrated composites' external metal layers consisted of abutting Cu domains without any applied strain and consisted of separated Cu domains upon the application of strain, indicating that the previously reported operating mechanism was maintained [Fig. 4(c)].^24–26^ These experiments demonstrated the straightforward fabrication of fabric-integrated composite materials with large areas, robust mechanical properties, and reconfigurable surface microstructures.

We next assessed the adaptive infrared properties of the fabric-integrated composite materials, as shown in Fig. 4(a). For this purpose, we characterized the composite materials with FTIR transmittance and reflectance spectroscopy according to established protocols (see Methods for additional information). The total infrared reflectance spectra obtained for the composites indicated average values that progressively decreased from ∼97% ± 1% to ∼79% ± 1% to ∼72% ± 1% under applied strains of 0%, 30%, and 50%, respectively [Fig. 4(d)]. The total infrared transmittance spectra obtained for the composites indicated average values that progressively increased from ∼1% ± 1% to ∼4% ± 1% to ∼7% ± 1% under applied strains of 0%, 30%, and 50%, respectively [Fig. 4(e)]. Here, the average transmittance changes observed for the mesh-integrated composite materials were smaller than those reported for the analogous composite materials without fabric integration (see the direct comparison in supplementary material Table 2), presumably because the transmission of infrared radiation through the adhered fabric featured a comparatively weak dependence on the applied strain (supplementary material Fig. 11).^25^ Notably, the mesh-integrated composites' average reflectance and transmittance modulation remained relatively consistent even after 1000, 5000, and 10 000 mechanical actuation cycles (supplementary material Fig. 12). These experiments showed that our composite materials' user-tunable infrared-reflecting and infrared-transmitting functionalities were generally maintained even after fabric integration.

We, in turn, assessed the dynamic thermoregulatory functionalities of the fabric-integrated composite materials, as shown in Fig. 4(a). For this purpose, we characterized the composite materials with calibrated heat flux measurements on a SGHP according to established protocols (see Methods for additional information). The plot of the time-dependent heat fluxes obtained for a representative mesh-integrated composite indicated a value of ∼190 W/m^2^ under an applied strain of 0% and a value of ∼204 W/m^2^ under an applied strain of 30% [Fig. 4(f)]. The average steady-state heat flux changes measured for such composites could be readily adjusted between ∼6 ± 2 and ∼14 ± 1 W/m^2^ by the applied strain [Fig. 4(g)]. Here, the heat flux changes observed for the mesh-integrated composite materials were again smaller than those reported for analogous composites without fabric integration (see the direct comparison in supplementary material Table 3), presumably because the flow of heat through the adhered fabric featured a comparatively weak dependence on the applied strain (supplementary material Fig. 11).^25^ These experiments showed that our composite materials' user-controllable heat-managing functionalities were generally maintained even after fabric integration.

We last comparatively benchmarked the adaptive infrared properties and dynamic heat-managing functionalities of the fabric-integrated composite materials. To achieve this goal, we compared the infrared reflectances, infrared transmittances, and heat fluxes for the mesh-integrated composites, analogous composites without fabric integration, and standalone meshes, as obtained from FTIR spectroscopy and SGHP measurements, respectively (see Methods for additional information). The average infrared reflectance changes measured for the mesh-integrated composites, analogous composites without fabric integration, and standalone meshes reached values of ∼25% ± 2%, ∼31% ± 3%, and ∼1% ± 2%, respectively, for applied strains of 50% (supplementary material Table 2).^25^ The average infrared transmittance changes measured for the mesh-integrated composites, analogous composites without fabric integration, and standalone meshes reached values of ∼6% ± 2%, ∼18% ± 2%, and ∼8% ± 2%, respectively, for applied strains of 50% (supplementary material Table 2).^25^ The heat flux changes measured for the mesh-integrated composites, analogous composites without fabric integration, and standalone meshes reached values of ∼14 ± 1, ∼29 ± 3, and ∼5 ± 1 W/m^2^, respectively, for applied strain of 30% (supplementary material Table 3).^25^ These experiments demonstrated that our composite materials could endow standard commercial meshes with adaptive infrared properties and dynamic thermoregulatory functionalities, suggesting that the overall integration approach would be generally applicable for other textiles.

### Preparation and evaluation of breathable, washable, and fabric-integrated composite materials

**FIG. 5.**
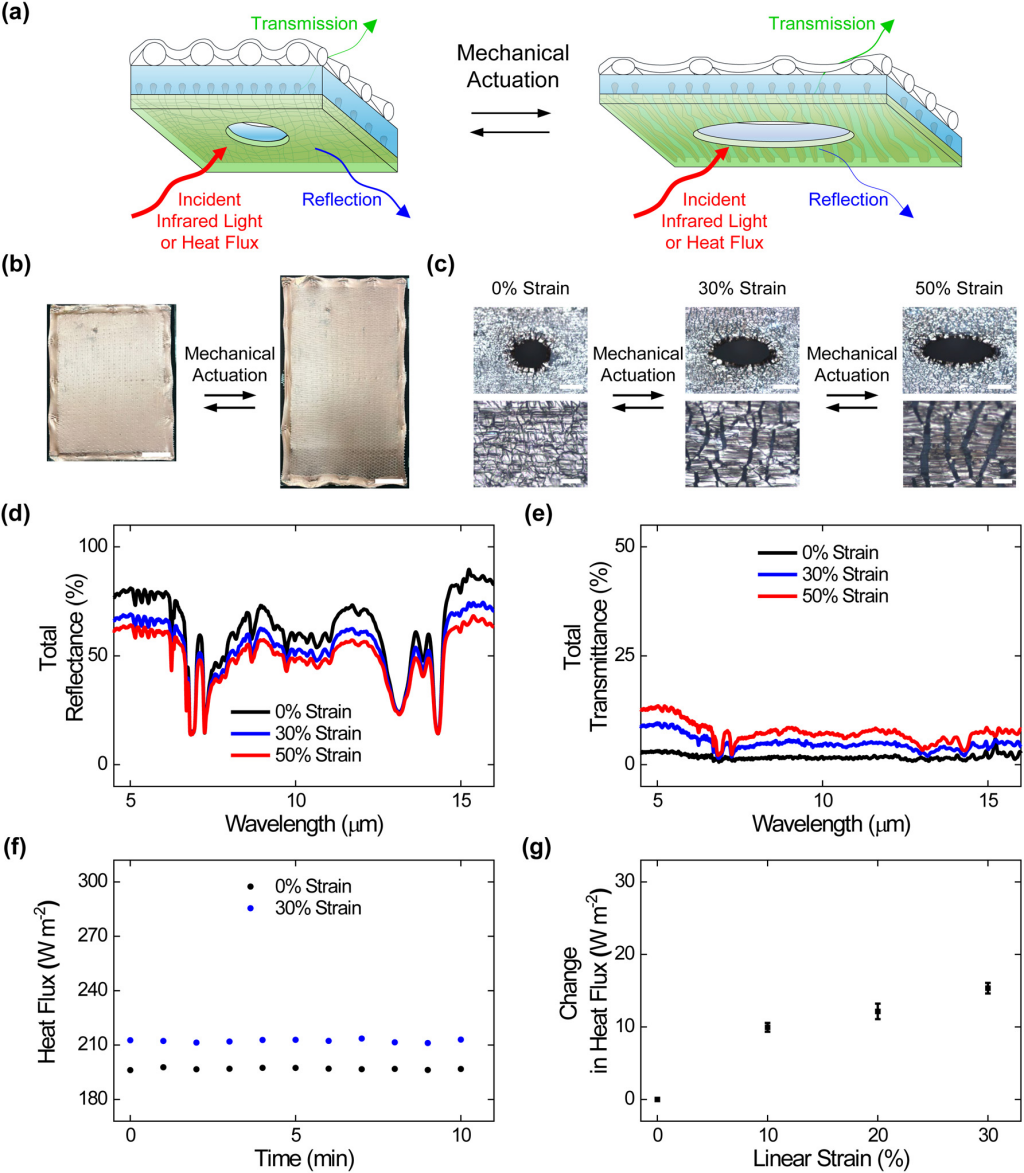
Surface morphologies, adaptive infrared properties, and dynamic thermoregulatory functionalities of the perforated, encapsulated, and fabric-integrated composite materials. (a) A schematic of a perforated, encapsulated, and fabric-integrated composite material that undergoes changes in surface microstructure and, thus, adaptively modulates infrared light and heat upon mechanical actuation. (b) Digital camera images of perforated, encapsulated, and fabric-integrated composites under applied strains of 0% (left) and 50% (right). The composites feature areas of ≥560 cm^2^. The scale bars are 4 cm. (c) High (top) and low (bottom) magnification optical microscopy images of the perforated, encapsulated, and fabric-integrated composites under applied strains of 0% (left), 30% (middle), and 50% (right). The images show that embedded metal islands transition between abutted and separated states upon the application of strain. The scale bars are 100 *μ*m for the top images and 20 *μ*m for the bottom images. (d) The total infrared reflectance spectra obtained for the perforated, encapsulated, and fabric-integrated composites under applied strains of 0% (black), 30% (blue), and 50% (red). (e) The total infrared transmittance spectra obtained for the perforated, encapsulated, and fabric-integrated composites under applied strains of 0% (black), 30% (blue), and 50% (red). (f) The plot of the time-dependent heat fluxes obtained for the perforated, encapsulated, and fabric-integrated composites under applied strains of 0% (black) and 30% (blue). (g) The average steady-state heat flux changes measured for the perforated, encapsulated, and fabric-integrated composites under applied strains of 0%, 10%, 20%, and 30%. The error bars in (g) represent standard deviation of the mean.

We completed our efforts by preparing perforated, encapsulated, and fabric-integrated composite materials and characterizing them without and with mechanical strain, as illustrated in Figs. 1(g) and 5(a). To this end, we fabricated large-area composites that contained arrayed holes, featured an SEBS encapsulation layer, and incorporated a commercial mesh, as illustrated in supplementary material Fig. 13(a), and we interrogated the perforated, encapsulated, and mesh-integrated composites with digital camera imaging, optical microscopy, and tensile testing (see Methods for additional information). The digital camera images revealed that the obtained perforated, encapsulated, and mesh-integrated composites featured areas of ≥560 cm^2^, were covered by ∼200 *μ*m holes with edge-to-edge separations of ∼1 mm, were completely enclosed by the encapsulation layer, were uniformly attached to the mesh, and were readily deformed via applied strain [Fig. 5(b) and supplementary material Fig. 13(b)]. The engineering stress vs strain curves and corresponding camera images indicated that the perforated, encapsulated, and mesh-integrated composites featured elastomeric behavior, with Young's moduli of ∼1.0 MPa and breaking strains of ∼340% confirming expectations from measurements for our other composite types (*vide supra*) (supplementary material Fig. 14 and supplementary material Table 1).^24–26^ The local optical microscopy images revealed that the perforated, encapsulated, and mesh-integrated composites' internal metal layers contained round holes surrounded by abutting Cu domains without any applied strain but contained oval holes surrounded by separated Cu domains upon the application of strain, confirming the operating mechanism expected from measurements for our other composite types (*vide supra*) [Fig. 5(c)].^24–26^ These experiments demonstrated the straightforward fabrication of multifunctional composite materials, which maintained their large areas, robust mechanical properties, and reconfigurable surface microstructures.

We next assessed the adaptive infrared properties of the perforated, encapsulated, and fabric-integrated composite materials, as shown in Fig. 5(a). For this purpose, we characterized such multifunctional composite materials with FTIR transmittance and reflectance spectroscopy according to established protocols (see Methods for additional information). The total infrared reflectance spectra obtained for the composites indicated average values that progressively decreased from ∼63% ± 1% to ∼55% ± 1% to ∼51% ± 1% under applied strains of 0%, 30%, and 50%, respectively [Fig. 5(d)]. The total infrared transmittance spectra obtained for the composites indicated average values that progressively increased from ∼2% ± 1% to ∼5% ± 1% to ∼7% ± 1% under applied strains of 0%, 30%, and 50%, respectively [Fig. 5(e)]. Here, the average reflectance and transmittance changes observed for the perforated, encapsulated, and mesh-integrated composite materials were smaller than those reported for analogous composites without any perforation, encapsulation, and fabric integration, in agreement with expectations for the combined influence of the arrayed holes, the SEBS-based encapsulation layer, and the adhered mesh (*vide supra*) (see the direct comparisons in supplementary material Table 2).^25^ Notably, the perforated, encapsulated, and mesh-integrated composites' average reflectance and transmittance modulation remained relatively consistent even after 1000, 5000, and 10 000 mechanical actuation cycles (supplementary material Fig. 15). These experiments showed that our multifunctional composite materials' user-tunable infrared-reflecting and infrared-transmitting functionalities were generally maintained even after the simultaneous modifications of perforation, encapsulation, and fabric integration.

We, in turn, assessed the dynamic thermoregulatory functionalities of the perforated, encapsulated, and fabric-integrated composite materials, as shown in Fig. 5(a). For this purpose, we characterized such multifunctional composite materials with calibrated heat flux measurements on a SGHP according to established protocols (see Methods for additional information). The plot of the time-dependent heat fluxes obtained for a representative perforated, encapsulated, and mesh-integrated composite indicated a value of ∼197 W/m^2^ under an applied strain of 0% and a value of ∼212 W/m^2^ under an applied strain of 30% [Fig. 5(f)]. The average steady-state heat flux changes measured for such composites could be readily adjusted between ∼9 ± 1 and ∼15 ± 1 W/m^2^ by the applied strain [Fig. 5(g)]. Here, the heat flux changes observed for the perforated, encapsulated, and mesh-integrated composite materials were again smaller than those reported for analogous composites without any perforation, encapsulation, and fabric integration, in agreement with expectations for the influence of the arrayed holes, the SEBS-based encapsulation layer, and the adhered fabric (*vide supra*) (see the direct comparisons in supplementary material Table 3).^25^ These experiments showed that our multifunctional composite materials' user-controllable heat-managing functionalities were generally maintained even after the simultaneous modifications of perforation, encapsulation, and fabric integration.

We last comparatively evaluated the adaptive infrared properties, dynamic thermoregulatory functionalities, air and water vapor permeabilities, and wash stabilities of the perforated, encapsulated, and fabric-integrated composite materials. To achieve this goal, we characterized the multifunctional composite materials with (1) FTIR transmittance and reflectance spectroscopy, (2) calibrated heat flux measurements, (3) standard air and water vapor permeability measurements, and (4) digital camera imaging and FTIR spectroscopy across multiple wash/dry cycles (see Methods for additional information). First, the infrared reflectance and infrared transmittance changes measured for the multifunctional composites reached values of ∼12% ± 2% and ∼5% ± 2%, respectively, for applied strains of 50%, resembling the trends measured for our various modified composites (supplementary material Table 2). Second, the heat flux changes measured for the multifunctional composites reached a value of ∼15 ± 1 W/m^2^ at 30% strain, resembling the trends measured for our various modified composites (supplementary material Table 3). Third, the air and water vapor permeabilities obtained for the multifunctional composites were ∼55 ± 3 ft^3^/ft^2^/min and ∼855 ± 20 g/m^2^/day, respectively, matching observations for our perforated composites (supplementary material Table 4). Finally, the visible appearances and infrared spectra recorded for the multifunctional composites remained nearly unchanged across 20 wash/dry cycles, matching observations for our encapsulated composites (supplementary material Figs. 7 and 16). These combined experiments demonstrated that our multifunctional composite materials not only maintained the adaptive infrared properties and dynamic thermoregulatory functionalities of the other composite types but also incorporated their multiple synergistic advantageous characteristics.

## CONCLUSION

In summary, we have prepared and studied breathable, washable, and/or fabric-integrated squid skin-inspired thermoregulatory composite materials, and our findings represent a significant advance for such materials for multiple reasons. First, the modified composites were all manufactured by using reported fabrication protocols that incorporated a minimal number of straightforward additional steps, i.e., perforation, lamination, and/or adhesion.^25^ Second, the modified composites all preserved not only their favorable mechanical properties, i.e., low Young's moduli and large breaking strains, but also their working mechanisms, i.e., strain-reconfigurable surface microstructures.^24–26^ Third, the described modified composites all generally maintained their adaptive infrared properties and dynamic thermoregulatory functionalities, i.e., the ability to modulate infrared reflectance, infrared transmittance, and heat flow.^24–26^ Here, we note that additional optimization of the perforation, lamination, and adhesion steps during fabrication could facilitate further improvement of the composites' operational performances. As such, the described findings appear to constitute a critical technological leap forward for the bioinspired composite materials.

For our composites, the reported breathabilities, wash stabilities, and fabric compatibilities may provide new opportunities within the context of wearable technologies. For example, the high air and water vapor permeabilities of the reported perforated composites could enable the development of more sophisticated heat-managing systems that simultaneously harness radiate and convective effects. Additionally, the excellent wash stabilities of the reported encapsulated composites seemingly remove limitations on their practical implementation in the many types of clothing that require routine and repeated cleaning. Furthermore, the good fabric compatibilities of the reported mesh-integrated composites suggest that their adaptive infrared properties and dynamic thermoregulatory functionalities could be incorporated into a broad range of textiles. Last, we believe that such promising opportunities are further underscored by the demonstration of multifunctional (breathable, washable, and mesh-integrated) composite materials. When considered together, our findings appear to substantially broaden the applications scope of the bioinspired composite materials.

More generally, the strategies that enabled breathable, washable, and fabric-integrated variants of our composite materials could be employed for other types of wearable systems. For instance, the laser cutting-based perforation technique used for enhancing our composites' breathabilities could be translated to stretchable and washable organic light-emitting diodes (OLEDs) or organic photovoltaics (OPVs), affording analogously increased breathabilities without sacrificing wash stability or degrading the devices' optoelectronic properties.^33–36^ In addition, the lamination-based encapsulation technique used for ensuring our composites' wash stabilities could be applied to stretchable e-textiles, affording improved waterproofing for delicate electronic components while maintaining the systems' mechanical properties.^37–39^ Moreover, the adhesive-based mesh integration technique used for demonstrating our composites' fabric compatibilities could be leveraged for flexible energy-harvesting triboelectric materials, facilitating form factor flexibility during fabric integration and thus minimally influencing functional performance.^39–42^ Given such possibilities, our findings appear well positioned to guide the incorporation of high-desirable multifunctionality into many other emerging wearable technologies.

## METHODS

### Fabrication of the perforated composite materials

The perforated composite materials were fabricated by incorporating an extra laser cutting step into the reported fabrication protocols, as illustrated in supplementary material Fig. 1.^25^ First, the standard composite materials with sizes of ∼28 × ∼20 cm^2^ (∼11 × ∼8 in.) were fabricated via a process that involved deposition of Cu nanostructured films, spray coating of stretchable polymer matrices, and manual delamination of the completed structure.^25^ Second, the free-standing standard composite materials with sizes of ∼28 × ∼20 cm^2^ (∼11 × ∼8 in.) were mounted on a home-built stage placed on the working table of a laser engraving system (Speedy 360, Trotec). Second, a laser cutting head was positioned in the middle of the standard composite materials under automated control (JobControl Expert, Trotec). Third, the perforated composite materials with sizes of ∼28 × ∼20 cm^2^ (∼11 × ∼8 in.) were obtained by using a CO_2_ laser (power of 48 W, velocity of 43 mm/s, frequency of 2 kHz, and resolution of 1000 dpi) to cut ∼200 *μ*m holes with edge-to-edge separations of ∼1 mm. Note that the chosen hole sizes and separations were optimized empirically to ensure mechanical integrity. The resulting perforated composite materials were used for the digital camera imaging, optical microscopy imaging, tensile testing, infrared spectroscopy, mechanical stability testing, thermal characterization, air permeability, and water vapor transmission experiments.

### Fabrication of the encapsulated composite materials

The encapsulated composite materials were fabricated by incorporating an extra lamination step into the reported fabrication protocols, as illustrated in supplementary material Fig. 4.^25^ First, the standard composite materials with sizes of ∼28 × ∼20 cm^2^ (∼11 × ∼8 in.) were fabricated via a process that involved deposition of Cu nanostructured films, spray coating of stretchable polymer matrices, and manual delamination of the completed structure.^25^ Second, the free-standing encapsulation layers with sizes of ∼28 × ∼20 cm^2^ (∼11 × ∼8 in.) were prepared by initial spray-coating of 5% (w/w) solutions of a SEBS block copolymer (G1645, Kraton) in toluene (Fisher Chemical) on a glass support substrate using an airbrushing system (1/5 HP Professional, Vivohome) installed on a three-dimensional printer (System 30M, Hyrel 3D) and by subsequent manual delamination from the support substrate. Third, the encapsulation layers were manually positioned on the Cu sides of the free-standing standard composite materials with sizes of ∼28 × ∼20 cm^2^ (∼11 × ∼8 in.). Fourth, the encapsulated composite materials with sizes of ∼28 × ∼20 cm^2^ (∼11 × ∼8 in.) were obtained by using a film sealing roller (MSR0001, MJ Research) to laminate the encapsulation layers onto the standard composite materials. The resulting encapsulated composite materials were used for the digital camera imaging, optical microscopy imaging, tensile testing, infrared spectroscopy, mechanical stability testing, thermal characterization, and wash stability testing experiments.

### Fabrication of the fabric-integrated composite materials

The fabric-integrated composite materials were fabricated by incorporating an extra mesh adhesion step into the reported fabrication protocols, as illustrated in supplementary material Fig. 9.^25^ First, the standard composite materials with sizes of ∼28 × ∼20 cm^2^ (∼11 × ∼8 in.) were fabricated via a process that involved deposition of Cu nanostructured films, spray coating of stretchable polymer matrices, and manual delamination of the completed structure.^25^ Second, the commercial meshes (R11W, APEX) with sizes of ∼28 × ∼20 cm^2^ (∼11 × ∼8 in.) were overlaid on top of the SEBS block copolymer side of the free-standing standard composite materials. Third, a 45% (w/w) adhesive solution of a SEBS block copolymer (G1645, Kraton) in toluene was applied to the interfaces between the meshes and the standard composite materials by using a 1 *μ*m hand-held manual syringe dispenser (Micro-Dot, Dymax). Fourth, the mesh-integrated composite materials with sizes of ∼28 × ∼20 cm (∼11 × ∼8 in) were obtained by allowing residual toluene solvent to evaporate in ambient atmosphere. The resulting fabric-integrated composite materials were used for digital camera imaging, optical microscopy imaging, tensile testing, infrared spectroscopy, mechanical stability testing, and thermal characterization experiments.

### Fabrication of the perforated, encapsulated, and fabric-integrated composite materials

The perforated, encapsulated, and fabricated-integrated composite materials were fabricated by incorporating extra encapsulation, laser cutting, and mesh adhesion steps into the reported fabrication protocols, as illustrated in supplementary material Fig. 13.^25^ First, the standard composite materials with sizes of ∼28 × ∼20 cm^2^ (∼11 × ∼8 in.) were fabricated via a process that involved deposition of Cu nanostructured films, spray coating of stretchable polymer matrices, and manual delamination of the completed structure.^25^ Second, the free-standing encapsulation layers were manually positioned on the Cu sides of the free-standing standard composite materials. The encapsulated composite materials with sizes of ∼28 × ∼20 cm^2^ (∼11 × ∼8 in.) were obtained by using a film sealing roller (MSR0001, MJ Research) to laminate the encapsulation layers onto the standard composite materials. Third, a laser cutting head was positioned in the middle of encapsulated composite materials mounted on a home-built stage placed on the working table of a laser engraving system (Speedy 360, Trotec) under automated control (JobControl Expert, Trotec). The perforated and encapsulated composite materials with sizes of ∼28 × ∼20 cm^2^ (∼11 × ∼8 in.) were obtained by using a CO_2_ laser (power of 48 W, velocity of 43 mm/s, frequency of 2 kHz, and resolution of 1000 dpi) to cut ∼200 *μ*m holes with edge-to-edge separations of ∼1 mm into the encapsulated composite materials. Fourth, the commercial meshes (R11W, APEX) were overlaid on the polymer matrix sides of the perforated and encapsulated composite materials and a 45% (w/w) adhesive solution of a SEBS block copolymer (G1645, Kraton) in toluene was applied to the interfaces between the meshes and the perforated and encapsulated composite materials by using a 1 *μ*m hand-held manual syringe dispenser (Micro-Dot, Dymax). The perforated, encapsulated, and mesh-integrated composite materials with sizes of ∼28 × ∼20 cm^2^ (∼11 × ∼8 in.) were obtained by allowing residual toluene solvent to evaporate in ambient atmosphere. The perforated, encapsulated, and fabric-integrated composite materials were used for digital camera imaging, optical microscopy imaging, tensile testing, infrared spectroscopy, mechanical stability testing, thermal characterization, air permeability, water vapor transmission, and wash stability testing experiments.

### Digital camera imaging of the composite materials

The visible appearance of the composite materials was characterized in house by using a digital camera (PowerShot SX520, Canon) according to established protocols.^43^ The composite materials were mounted on clear polyester film (Dura-Lar, Grafix) frames with electrical tape (165 Temflex, 3M) prior to imaging. The digital camera images were obtained for the composite materials on a flat working table. The pictures were analyzed by the Photoshop (Adobe) software package. The digital camera imaging experiments were performed for a minimum of three independent perforated composites, encapsulated composites, mesh-integrated composites, and perforated, encapsulated, and mesh-integrated composites.

### Optical microscopy imaging of the composite materials

The morphologies of the composite materials were characterized in house by using an optical microscope (Axio Imager.A2m, Zeiss) according to established protocols.^44^ The composite materials were subjected to the desired strains of 0, 30, or 50% when mounted on a microscope slide (Premium Microscope Slides Plain, Fisher Scientific) with a total size of 3 × 1. The optical microscopy images were obtained at typical magnifications of 10× and 50×. The optical microscopy images were analyzed by the Photoshop (Adobe) software package. The optical microscopy imaging experiments were performed for a minimum of three independent perforated composites, encapsulated composites, mesh-integrated composites, and perforated, encapsulated, and mesh-integrated composites.

### Tensile testing of the composite materials

The mechanical properties of the composite materials were characterized in house by using a Tensile Testing System (3365 Universal, Instron) according to established protocols.^45^ The composite materials were fixed by the pneumatic side action tensile grips of the system and actuated three times between 0 and 100% uniaxial strain at a rate of 15 mm/s to ensure reproducibility prior to testing.^45^ The engineering stress vs strain curves were obtained by stretching the composite materials from 0% strains to their breaking strains at rates of 30 mm/s. The Young's moduli were calculated from the linear regions of the engineering stress vs strain curves at a strain value of 30%. The average Young's moduli were analyzed with the Origin 8.5 (OriginLab) software packages. The tensile testing experiments were performed for a minimum of three independent perforated composites, encapsulated composites, mesh-integrated composites, standalone meshes, and perforated, encapsulated, and mesh-integrated composites.

### Infrared spectroscopy of the composite materials

The infrared functionalities of the materials were characterized in house by Fourier transform infrared (FTIR) transmittance and reflectance spectroscopy using a FTIR spectrometer (Frontier, PerkinElmer) outfitted with a mid-infrared integrating sphere (Mid-IR IntegratIR, Pike Technologies) according to established protocols.^46^ The materials were mounted on home-built size-adjustable stages and were mechanically actuated by strains of 0%, 30%, or 50%. The composite materials were large enough to completely cover the port of the instrument both before and after mechanical actuation. The total reflectance spectra were obtained for the materials under various strains at a 12° illumination angle, and the total transmittance spectra were obtained under various strains at a normal illumination angle. The average total reflectance and total transmittance values were calculated from the spectra over a wavelength range of 4.5–16.5 *μ*m. The spectra were analyzed with the Spectrum (PerkinElmer) and Origin 8.5 (OriginLab) software packages. The infrared spectroscopy experiments were performed for a minimum of three independent perforated composites, encapsulated composites, mesh-integrated composites, standalone meshes, and perforated, encapsulated, and mesh-integrated composites.

### Mechanical stability testing of the composite materials

The stability of the composite materials was characterized in house by a combination of mechanical cycling and FTIR spectroscopy.^25,46^ The composite materials were mounted on a tension/compression test stand (ESM303, MARK-10) and were mechanically cycled 0, 1000, 5000, or 10 000 times between applied uniaxial strains of 0% and 50% at a frequency of 1 Hz by using the tension/compression test stand. The total reflectance and total transmittance spectra were obtained for the materials under an applied strain of 0%, 30%, or 50% after 0, 1000, 5000, or 10000 mechanical actuation cycles by using a FTIR spectrometer (Frontier, PerkinElmer). The average total reflectance and total transmittance values were calculated from the spectra over a wavelength range of 4.5–16.5 *μ*m. The spectra were analyzed with the Spectrum (PerkinElmer) and Origin 8.5 (OriginLab) software packages. The stability testing experiments were performed for a minimum of three independent perforated composites, encapsulated composites, mesh-integrated composites, and perforated, encapsulated, and mesh-integrated composites.

### Thermal characterization of the composite materials

The thermal properties of the composite materials were characterized in house by using a sweating guarded hot plate (SGHP) installed in a custom chamber (SGHP-8.2, Thermetrics) according to established protocols.^47^ The materials were mounted on a home-built size-adjustable holder and were mechanically actuated by strains of 0%, 10%, 20%, or 30%. The time-dependent heat flux was measured for the materials under various strains at a hot plate temperature of 35 °C, chamber temperature of 19.5 °C, chamber relative humidity of 50%, and laminar airflow velocity of 1 m/s. The average heat flux and heat flux change values were calculated from the heat flux values recorded as a function of time. The heat flux and heat flux change values were analyzed with the Origin 8.5 (OriginLab) software package. The thermal characterization experiments were performed for a minimum of three independent perforated composites, encapsulated composites, mesh-integrated composites, standalone meshes, and perforated, encapsulated, and mesh-integrated composites.

### Air permeability testing of the composite materials

The air permeabilities of the composite materials were characterized at Precision Testing Laboratories by using an air permeability tester (FX3300 LabAir IV, TexTest) according to established protocols.^48^ The materials were mounted on the test head opening with the clamping arm of the tester, and a suction pump installed under the test head opening enabled air to be forced through the materials. The air permeabilities were obtained for the materials by measuring the air flow resistance at a test pressure of 124 Pa. The air permeability values were calculated and analyzed with the Origin 8.5 (OriginLab) software package. The air permeability characterization experiments were performed for a minimum of three independent standard composites, perforated composites, and perforated, encapsulated, and mesh-integrated composites.

### Water vapor transmission testing of the composite materials

The water vapor permeabilities of the composite materials were characterized at Precision Testing Laboratories by using a 1056 environmental chamber (Precision Testing Laboratories) according to established protocols.^49^ The materials were mounted on a cup filled with distilled water, covered with a gasket, and placed in an equilibrated environmental chamber. The water vapor permeabilities were obtained for the materials by calculating the amount of water evaporated from the cup after 24 h at a chamber temperature of 23 °C, relative humidity of 50%, and laminar air flow velocity of 0.3 m/s. The water vapor permeability values were calculated and analyzed with the Origin 8.5 (OriginLab) software package. The water vapor transmission experiments were performed for a minimum of three independent standard composites, perforated composites, and perforated, encapsulated, and mesh-integrated composites.

### Wash stability testing of the composite materials

The wash stabilities of the composite materials were characterized by a combination of digital camera imaging^43^ and FTIR spectroscopy^46^ across multiple wash cycles performed according to established protocols.^50,51^ The composite materials were placed into a commercial washing machine (HLP21N, Haier) along with 15 g of detergent (Acti-Lift, Tide). The materials were then subjected to 0, 1, 10, and 20 standard wash/dry cycles, which included washing, rinsing, and spinning phases lasting ∼35 min and a line drying phase lasting ∼30 min. The digital camera images were obtained for the composite materials after 0, 1, 10, and 20 cycles using a digital camera (PowerShot SX520, Canon). The total reflectance and total transmittance spectra were obtained for the composite materials under applied strains of 0%, 30%, and 50% after 0, 1, 10, and 20 wash cycles using the FTIR spectrometer (Frontier, PerkinElmer). The digital camera images were analyzed by the Photoshop (Adobe) software package, and the spectra were analyzed with the Spectrum (PerkinElmer) and Origin 8.5 (OriginLab) software packages. The wash stability characterization experiments were performed for a minimum of three independent standard composites, encapsulated composites, and perforated, encapsulated, and mesh-integrated composites.

## SUPPLEMENTARY MATERIAL

See the supplementary material for the details regarding fabrication schemes for the modified composite materials (see supplementary material Figs. 1, 4, 9, and 13); the engineering stress vs strain curves for the modified composites (see supplementary material Figs. 2, 5, 10, and 14); the mechanical stability characterization for the modified composites (see supplementary material Figs. 3, 6, 12, and 15); the wash stabilities of the standard and modified composites (see supplementary material Figs. 7, 8, and 16); the adaptive infrared properties and dynamic thermoregulatory functionalities of the standalone meshes (see supplementary material Fig. 11); the tabulated Young's moduli and breaking strains for the standard and modified composites (see supplementary material Table 1); the tabulated infrared reflectances/transmittances and infrared reflectance/transmittance changes for the standard and modified composites (see supplementary material Table 2); the tabulated heat fluxes and heat flux changes for the standard and modified composites (see supplementary material Table 3); and the tabulated air and water vapor permeabilities for the standard and modified composites (see supplementary material Table 4).

## Data Availability

The data that support the findings of this study are available within the article and its supplementary material.
